# Applications of the new ESPEN definition of malnutrition and SARC-F in Chinese nursing home residents

**DOI:** 10.1038/s41598-018-33350-w

**Published:** 2018-10-08

**Authors:** Ming Yang, Zhaojing Huang, Jing Chen, Jiaojiao Jiang, Yun Zuo, Qiukui Hao

**Affiliations:** 1 The Center of Gerontology and Geriatrics, West China Hospital, Sichuan University, No. 37 Guoxue Lane, Chengdu, Sichuan China; 2 The Center of Rehabilitation, West China Hospital, Sichuan University, No. 37 Guoxue Lane, Chengdu, Sichuan China; 3 The Health Management Center, Shangjin Nanfu Hospital, No. 253 Shangjin Street, Chengdu, Sichuan China

## Abstract

We aimed to compare the predictive capacity of malnutrition, sarcopenia, and malnutrition combined with sarcopenia for mortality in nursing home residents. We conducted a prospective study in four nursing homes in China. Nutrition status and sarcopenia were measured according to the new European Society of Clinical Nutrition and Metabolism (ESPEN) definition and SARC-F, respectively. The study population was divided into four groups: malnutrition with sarcopenia (MN+/SA+), malnutrition without sarcopenia (MN+/SA−), sarcopenia without malnutrition (MN−/SA+), and normal nutrition without sarcopenia (MN−/SA−). The participants were followed up for 12 months. We included 329 participants. Thirty-eight participants (11.6%) had MN+/SA+, 38 participants (11.6%) had MN+/SA−, and 93 participants (28.3%) had MN−/SA+. The 1-year mortality was 18.3%, 21.5%, 18.4%, and 47.4% in the MN−/SA−, MN−/SA+, MN+/SA−, and MN+/SA+ groups, respectively. Compared to participants with MN−/SA−, participants with MN+/SA+ were at a significantly higher risk of mortality (adjusted hazard ratio [HR]: 3.19, 95% confidence interval [CI] 1.71–5.95); however, MN−/SA+ (adjusted HR: 1.24, 95% CI 0.69–2.22) and MN+/SA− (adjusted HR: 0.95, 95% CI 0.41–2.19) were not predictors of mortality. In conclusion, the coexistence of malnutrition and sarcopenia is a significant predictor of mortality in a study population of Chinese nursing home residents.

## Introduction

Malnutrition refers to the imbalance between nutrition intake and requirements that ultimately causes the changes in body weight, body composition, and physical function^1^. The prevalence of malnutrition increases with aging, comorbidities, and levels of care^2^. Therefore, malnutrition is also considered as a geriatric syndrome^3^. Malnutrition is prevalent in elderly adults, especially in those living in nursing homes^4^. According to a recent review, approximately 20% of nursing home residents had malnutrition, but the prevalence ranged from 1.5% to 66.5% due to various definitions of malnutrition^4^. However, there is currently no data addressing malnutrition in nursing home residents in mainland China.

The lack of unique diagnostic criteria of malnutrition hinders the extensive research of it^5^. To address this issue, the European Society of Clinical Nutrition and Metabolism (ESPEN) published a new consensus statement on the diagnosis of malnutrition in 2015^5^. Since then, many studies have applied the new ESPEN definition of malnutrition in different clinical settings^6–14^. However, most of these studies were conducted in hospitals and no study applied the new ESPEN definition of malnutrition in nursing homes.

Sarcopenia is another geriatric syndrome that is highly prevalent in elderly adults, especially in hospitalized and institutionalized elders^15^. Sarcopenia refers to the age-related loss of muscle mass, strength, and performance^16^. The diagnosis of sarcopenia also lacks unique criteria, but it is device-dependent (e.g., computed tomography [CT], magnetic resonance imaging [MRI], dual-energy x-ray absorptiometry [DXA]), time-consuming, and sometimes inaccessible according to current international consensuses of sarcopenia^16–19^. A brief screening tool for sarcopenia is therefore needed, especially for nursing home residents. SARC-F, a classical sarcopenia screening tool, has been widely applied in various populations^20^. However, to our knowledge, SARC-F has not been applied to nursing home residents yet.

Both malnutrition and sarcopenia have been shown to be related to an increased risk of mortality and other adverse health outcomes, such as poor quality of life and functional impairments^4,21^. However, whether the new ESPEN definition of malnutrition or SARC-F can predict mortality in nursing home residents remains unclear. In addition, we hypothesized that nursing home residents with coexisting malnutrition and sarcopenia were at a higher risk of mortality compared to those with either malnutrition or sarcopenia alone.

Therefore, the aim of this study is (1) to investigate the prevalence of malnutrition, sarcopenia, and the coexistence of malnutrition and sarcopenia in a study population of Chinese elderly adults living in nursing homes according to the new ESPEN definition of malnutrition and SARC-F; and (2) to compare the predictive capacity of malnutrition, sarcopenia, and malnutrition combined with sarcopenia for mortality in this population.

## Methods

We conducted a prospective observational study in four nursing homes in Chengdu City, China. The study protocol was approved by the Research Ethics Committee of our university. A written informed consent was obtained from all participants (or their legal proxies for those who were unable to sign their names). All methods in this study were in accordance with relevant regulations and guidelines.

### Baseline study population

From September to November 2016, we consecutively recruited elderly adults (≥70 years of age) living in the nursing homes. The exclusion criteria were as follows: (1) living in the nursing homes less than two weeks; (2) unable to communicate with the interviewers; and (3) refusing to participate in this study.

### Data collection

Trained nurses collected the key baseline data through face-to-face interviews and performed the following anthropometric measurements: calf circumference (CC) and waist circumference (WC). The CC was measured using a flexible tape at its widest point when the subjects placed in the supine position, with their left knee raised and the calf placed at a right angle to the thigh. The WC was measured using a flexible tape at the top of the hip bone on the naked skin at the end of light exhalation with the subject standing. Additionally, body mass index (BMI) was collected from the medical records of the nursing homes.

### Nutrition status assessment

According to the new ESPEN criteria^5^, the diagnosis of malnutrition includes two steps: First, a validated risk screening tool is suggested to identify individuals at risk of malnutrition. In this study, trained nurses applied the Mini Nutritional Assessment Short-Form (MNA-SF)^22^ as the screening tool. A total score of MNA-SF ≤ 11 indicated that an individual was at risk of malnutrition^22^. Second, among the participants who were at risk of malnutrition by the MNA-SF, malnutrition was confirmed according to either of the following criteria: (1) BMI < 18.5 kg/m^2^; or (2) unintentional weight loss >10% indefinite of time (or >5% over the last 3 months) combined with BMI < 20 kg/m^2^ if age <70 years or <22 kg/m^2^ if age ≥70 years^5^.

### Sarcopenia screening

We applied the SARC-F to screen sarcopenia in this study. The SARC-F includes five items: strength, assistance in walking, rising from a chair, climbing stairs, and falls (Supplementary Table 1)^20^. A total score of SARC-F ≥ 4 indicates sarcopenia^20^.

### Study groups

First, we divided the study population into two groups according to malnutrition and sarcopenia, respectively. Second, considering malnutrition and sarcopenia together, we divided the study population into four groups: malnutrition with sarcopenia (MN+/SA+), malnutrition without sarcopenia (MN+/SA−), sarcopenia without malnutrition (MN−/SA+), and normal nutrition without sarcopenia MN−/SA−).

### Covariates

Trained nurses evaluated the activities of daily living (ADL) of the participants using the Physical Self-Maintenance Scale (PSMS) developed by Lawton and Brody^23^. ADL disability was defined as “need some help from other people” or “cannot do by myself” in any of the six items of PSMS: eating, dressing, grooming, walking, bathing, and toileting^23^.

In addition, the following data were obtained from the medical records in the nursing homes: age, gender, smoking status, alcohol drinking status, and comorbidities (hypertension, ischemic heart disease, chronic heart failure, chronic obstructive pulmonary disease, diabetes, stroke, cancer of any type, osteoarthritis, Parkinson’s disease, cognitive impairment, and depression).

### Follow-up

The survival status of the participants was collected via the medical records in the nursing homes at the 12^th^ month after the baseline investigation. For those who left the nursing homes, we collected the survival status via telephone interviews. Next, the time to death was calculated as the period between the baseline investigation and the date of death.

### Statistical analysis

We performed all statistical analyses using SPSS 20.0 (IBM SPSS Statistics, Armonk, NY, USA). A p value of <0.05 was considered statistically significant. The categorical variables in this study were presented as counts and percentages. The normality of the continuous variables was explored by the Kolmogorov-Smirnov test. The continuous variables were not normally distributed; therefore, they were presented as a median and interquartile range (IQR). For categorical variables, the difference between groups was compared using the Pearson chi-squared test. For continuous variables, the difference between groups was compared using the Kruskal-Wallis H test (for the comparisons across multiple groups) or Mann-Whitney U test (for the comparisons between two groups).

We applied univariate Cox proportional hazard models to explore the possible predictors of 1-year all-cause mortality. The results were presented as hazard ratio (HR) and 95% confidence intervals (CIs). We also applied multivariable Cox proportional hazard models with a backward stepwise selection to explore the independent predictors of mortality. Age, gender, and the variables that exhibited a significant association in univariate analysis were considered to enter the initial model of the backward stepwise selection. In these models, the criteria for entry and removal were p < 0.05 and p > 0.10, respectively. In these models, sarcopenia and malnutrition were treated as variables, separately. Furthermore, we treated sarcopenia combined with malnutrition as one variable in a new model. In addition, survival curves were estimated using the Kaplan-Meier method. The difference between the survival curves was compared using the log-rank test.

## Results

### Description of the study population

**Figure 1:**
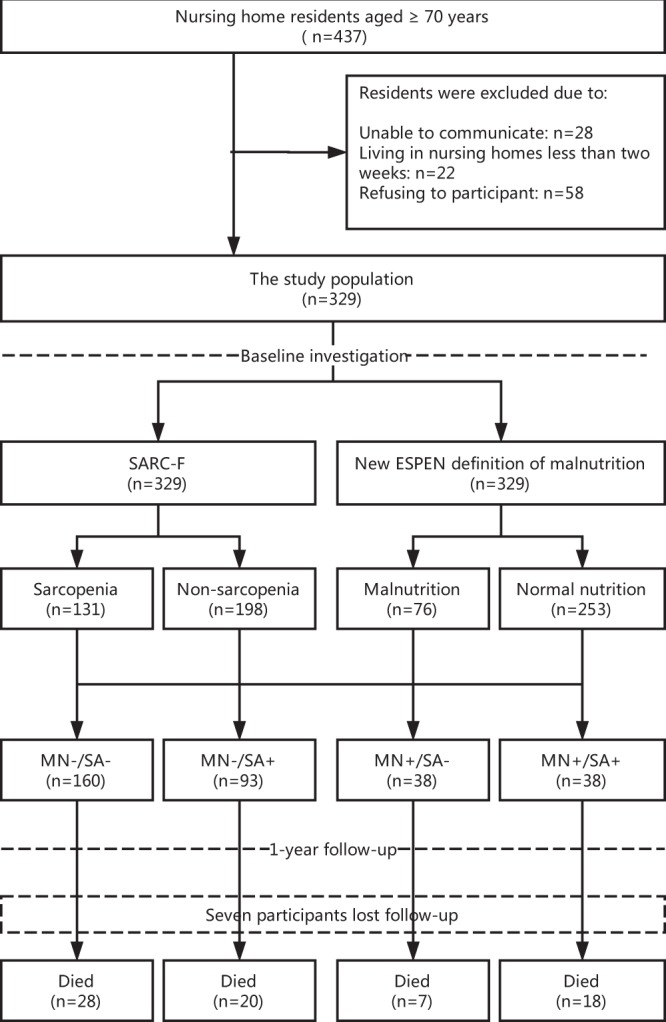
The study flow diagram.

We included 329 participants (105 men and 224 women) in the baseline investigation (Fig. 1). The median age of the whole study population was 85.0 years (men: 85.0 years; women: 85.0 years, p = 0.186). The baseline characteristics of the study population are shown in Table 1.

**Table 1:** Baseline characteristics of the study population according to malnutrition and sarcopenia.

Characteristic	Total (n = 329)	MN−/SA− (n = 160)	MN−/SA+ (n = 93)	MN+/SA− (n = 38)	MN+/SA+ (n = 38)	p
Age (years)^*^	85.0 (3.0)	85.0 (2.8)	84.0 (2.0)	85.0 (4.3)	85.0 (4.0)	0.003
Women (%)	224 (68.1)	100 (62.5)	69 (74.2)	26 (68.4)	29 (76.3)	0.166
Current smokers (%)	10 (3.0)	5 (3.1)	2 (2.2)	2 (5.3)	1 (2.6)	0.822
Current alcohol drinkers (%)	23 (7.0)	13 (8.1)	7 (7.5)	1 (2.6)	2 (5.3)	0.650
Comorbidities (%)						
Hypertension	99 (30.1)	50 (31.3)	35 (37.6)	6 (15.8)	8 (21.1)	0.051
Ischemic heart disease	33 (10.0)	14 (8.8)	13 (14.0)	0 (0)	6 (15.8)	0.057
CHF	112 (34.0)	54 (33.8)	32 (34.4)	9 (23.7)	17 (44.7)	0.288
COPD	43 (13.1)	17 (10.6)	19 (20.4)	4 (10.5)	3 (7.9)	0.094
Diabetes	33 (10.0)	19 (11.9)	9 (9.7)	3 (7.9)	2 (5.3)	0.623
Stroke	56 (17.0)	32 (20.0)	14 (15.1)	7 (18.4)	3 (7.9)	0.314
Cancer	26 (7.9)	15 (9.4)	8 (8.6)	1 (2.6)	2 (5.3)	0.502
Osteoarthritis	191 (58.1)	89 (55.6)	58 (62.4)	19 (50.0)	25 (65.8)	0.385
Parkinson’s disease	31 (9.4)	9 (5.6)	14 (15.1)	1 (2.6)	7 (18.4)	0.008
Cognitive impairment	71 (21.6)	36 (22.5)	13 (14.0)	12 (31.6)	10 (26.3)	0.111
Depression	68 (20.7)	31 (19.4)	23 (24.7)	5 (13.2)	9 (23.7)	0.454
Nutritional supplements (%)	51 (15.5)	22 (13.8)	17 (18.3)	6 (15.8)	6 (15.8)	0.819
SARC-F score^*^	3.0 (2.0)	2.0 (2.0)	5.0 (1.0)	2.0 (2.0)	5.0 (3.0)	<0.001
MNA-SF score^*^	11.0 (3.0)	13.0 (3.0)	13.0 (2.0)	10.0 (2.3)	9.0 (2.5)	<0.001
BMI (women, kg/m^2^)^*^	24.2 (4.1)	24.7 (3.6)	25.6 (3.5)	22.1 (1.6)	21.7 (3.1)	<0.001
BMI (men, kg/m^2^)^*^	23.9 (4.9)	24.8 (3.8)	24.3 (4.9)	20.5 (3.8)	19.7 (3.2)	<0.001
CC (women, cm)^*^	32.0 (3.0)	32.0 (4.0)	32.0 (2.0)	31.0 (2.0)	30.0 (3.0)	<0.001
CC (men, cm)^*^	33.0 (4.0)	34.0 (3.0)	33.0 (2.8)	31.5 (2.5)	29.0 (2.0)	<0.001
WC (women, cm)^*^	85.0 (12.8)	87.0 (11.5)	87.0 (10.0)	76.0 (9.3)	76.0 (11.0)	<0.001
WC (men, cm)^*^	86.0 (13.5)	88.0 (11.8)	86.0 (13.3)	77.0 (11.5)	70.0 (11.0)	<0.001
ADL disability (%)	138 (41.9)	62 (38.8)	37 (39.8)	12 (31.6)	27 (71.1)	0.001

^*^Data are presented as median (IQR).

The chi-square test was performed for categorical data and the Kruskal-Wallis H test for continuous data without normal distribution. P < 0.05 was considered statistically significant.

ADL: activities of daily living; BMI: body mass index; CC: calf circumference; CHF: chronic heart failure; COPD: chronic obstructive pulmonary disease; IQR: interquartile range; MNA-SF: Mini Nutritional Assessment Short-Form; MN+/SA+: malnutrition combined with sarcopenia; MN+/SA−: malnutrition without sarcopenia; MN−/SA+: normal nutrition with sarcopenia; MN−/SA−: normal nutrition without sarcopenia; WC: waist circumference.

### Characteristics of participants according to malnutrition and sarcopenia

According to the new ESPEN definition, 76 participants (23.1%) were malnourished. There was no significant difference between men and women with respect to malnutrition (20.0% versus 24.6%, p = 0.361). The characteristics of participants with or without malnutrition are presented in Supplementary Table 2. Not surprisingly, men or women with malnutrition had significantly lower medians of BMI, WC, and CC compared to their counterparts without malnutrition. In addition, hypertension was less prevalent in participants with malnutrition compared to those without malnutrition.

According to the SARC-F, 131 participants (39.8%) had sarcopenia. Women were more prone to sarcopenia than men (43.8% versus 31.4%, p = 0.033). The characteristics of participants with or without sarcopenia are presented in Supplementary Table 2. Parkinson’s disease and ischemic heart disease were more prevent in participants with sarcopenia than those without sarcopenia.

### Association between malnutrition and sarcopenia

Compared to those without malnutrition, participants with malnutrition had significantly higher SARC-F scores (median: 3.5 versus 3.0, p = 0.039) and were more prone to sarcopenia (50.0% versus 36.8%, p = 0.039). On the other hand, participants with sarcopenia had significantly lower MNA-SF scores (median: 11.0 versus 13.0, p = 0.025) and were more prone to malnutrition (29.0% versus 19.2%, p = 0.039) compared to those without sarcopenia.

When considering malnutrition and sarcopenia together, 38 participants (11.6%) suffered from MN+/SA+, 38 participants (11.6%) had MN+/SA−, and 93 participants (28.3%) had MN−/SA+.

### One-year mortality of the study population

During the 1-year follow-up, seven participants (2.1%) lost follow-up. A total of 73 participants (22.7%) died during the follow-up. The mortality was 23.5% in men and 22.2% in women (p = 0.752).

### Malnutrition and 1-year mortality

The 1-year mortality was 32.9% and 19.5% in participants with or without malnutrition, respectively (p = 0.015). Table 2 shows the predictors of 1-year mortality according to the Cox proportional hazard models. The univariate Cox proportional hazard model revealed that malnutrition was a predictor of 1-year mortality (HR: 1.79, 95% CI 1.10–2.90, p = 0.018).

**Table 2:** Predictors of 1-year all-cause mortality according to Cox proportional hazard models.

Variables	Univariate model^a^			Multivariate model^b^			Multivariate model^c^		
	HR	95% CI	P	HR	95% CI	P	HR	95% CI	P
Malnutrition	1.79	1.10–2.90	0.018	1.75	1.06–2.89	0.026	N/A	N/A	N/A
Sarcopenia	1.65	1.04–2.61	0.032	1.72	1.09–2.79	0.022	N/A	N/A	N/A
ADL disability	1.23	1.05–2.47	0.012	1.03	0.64–1.63	0.918	1.14	0.70–1.84	0.608
Age (per year)	1.03	0.97–1.10	0.349	1.02	0.95–1.09	0.600	1.03	0.96–1.10	0.344
Gender (women)	0.93	0.57–1.52	0.771	0.92	0.56–1.52	0.746	0.88	0.53–1.45	0.879
MN−/SA−	1 (Ref)	1 (Ref)	1 (Ref)	1 (Ref)	1 (Ref)	1 (Ref)	1 (Ref)	1 (Ref)	1 (Ref)
MN−/SA+	1.18	0.66–2.09	0.580	N/A	N/A	N/A	1.24	0.69–2.22	0.472
MN+/SA−	0.99	0.43–2.26	0.981	N/A	N/A	N/A	0.95	0.41–2.19	0.948
MN+/SA+	2.99	1.65–5.41	<0.001	N/A	N/A	N/A	3.19	1.71–5.95	<0.001

ADL: activities of daily living; MN+/SA+: malnutrition combined with sarcopenia; MN+/SA−: malnutrition without sarcopenia; MN−/SA+: normal nutrition with sarcopenia; MN−/SA−: normal nutrition without sarcopenia; N/A: not included in the models.

^a^Only significant variables are presented except for age and gender.

^b^Malnutrition and sarcopenia were treated as independent variables.

^c^Malnutrition combined with sarcopenia was treated as one variable.

**Figure 2:**
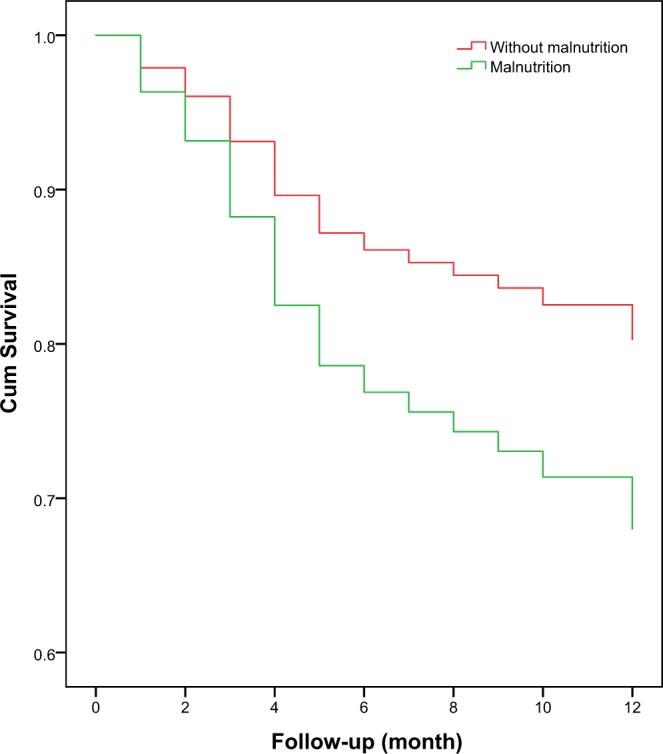
Survival curves of the study population according to nutrition status at baseline. Survival curves significantly differed in the log-rank test (p = 0.018).

After adjusted for the potential confounders, malnutrition was also independently associated with an increased risk of 1-year mortality (adjusted HR: 1.75, 95% CI 1.06–2.89, p = 0.026) (Table 2). The survival curves of the participants categorized by malnutrition are shown in Fig. 2. These survival curves were significantly different by the log-rank test (p = 0.012).

### Sarcopenia and 1-year mortality

**Figure 3:**
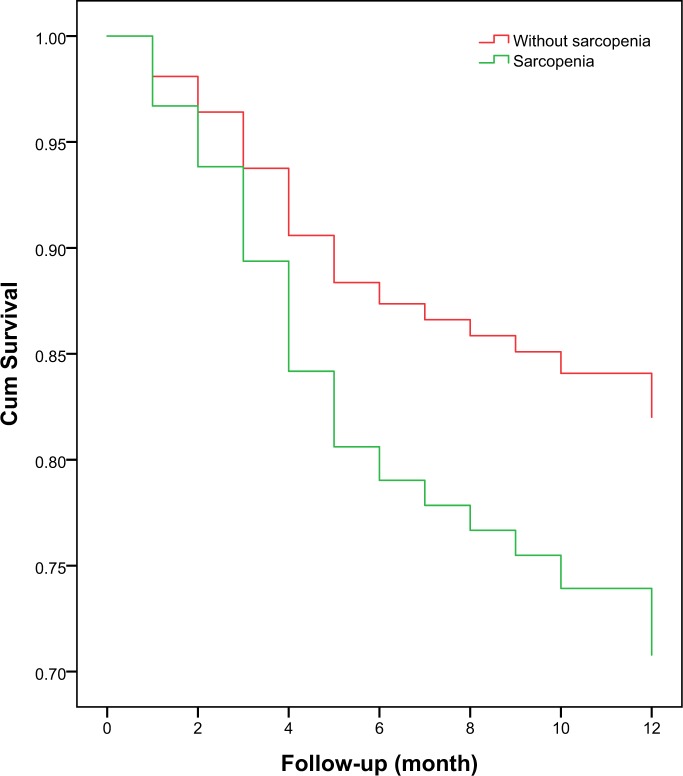
Survival curves of the study population according to sarcopenia at baseline. Survival curves significantly differed in the log-rank test (p = 0.020).

The 1-year mortality was 29.0% and 18.3% in the participants with or without sarcopenia, respectively (p = 0.025). The univariate Cox proportional hazard model revealed that sarcopenia was a predictor of 1-year mortality (HR: 1.65, 95% CI 1.04–2.61, p = 0.032). After adjusted for the potential confounders, sarcopenia was also independently associated with an increased risk of 1-year mortality (adjusted HR: 1.72, 95% CI 1.09–2.79, p = 0.022) (Table 2). The survival curves of the participants categorized by sarcopenia are shown in Fig. 3. These survival curves were significantly different by the log-rank test (p = 0.020).

### Malnutrition combined with sarcopenia and 1-year mortality

When considering malnutrition and sarcopenia together, the 1-year mortality was 18.3%, 21.5%, 18.4%, and 47.4% in the MN−/SA−, MN−/SA+, MN+/SA−, and MN+/SA+ groups, respectively. Compared to participants with MN−/SA−, participants with MN+/SA+ were at a significantly higher risk of 1-year mortality (adjusted HR: 3.19, 95% CI 1.71–5.95, p < 0.001); however, MN−/SA+(adjusted HR: 1.24, 95% CI 0.69–2.22, p = 0.472) and MN+/SA− (adjusted HR: 0.95, 95% CI 0.41–2.19, p = 0.948) were not predictors of mortality (Table 2).

**Figure 4:**
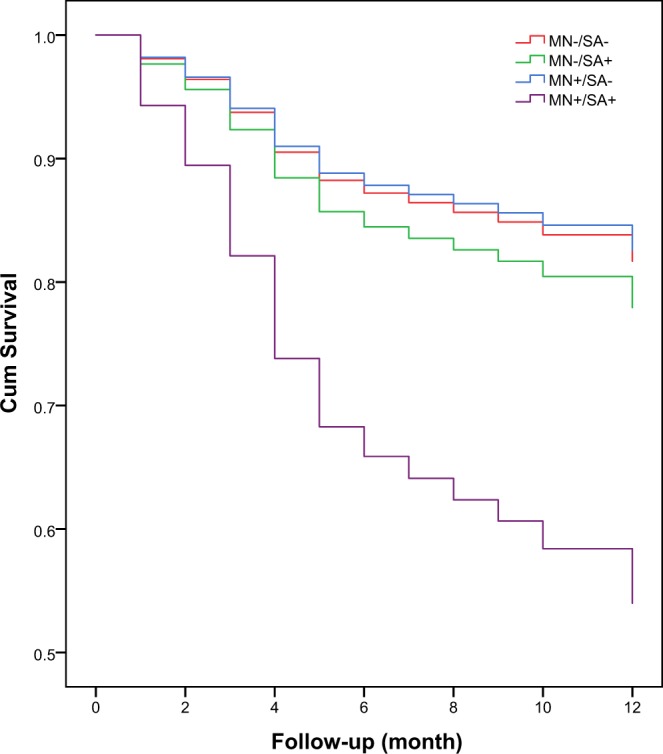
Survival curves of the study population according to sarcopenia and nutrition status at baseline. Survival curves significantly differed in the log-rank test (p < 0.001).

In addition, the survival curves of the participants categorized by sarcopenia are shown in Fig. 4. These survival curves were significantly different by the log-rank test (p < 0.001).

## Discussion

To the best of our knowledge, this study is the first to apply the new ESPEN definition of malnutrition and SARC-F in nursing home residents. It is also the first study to address malnutrition and sarcopenia in Chinese nursing home residents. Our study indicates that both the new ESPEN definition of malnutrition and SARC-F can be successfully applied to nursing home residents. Both tools can predict 1-year mortality in our study population. More importantly, participants with coexisting malnutrition and sarcopenia (MN+/SA+) were at significantly higher risk of mortality; however, sarcopenia alone (MN−/SA+) or malnutrition alone (MN+/SA−) did not increase the risk of mortality.

The aim of the new ESPEN consensus on malnutrition is to “provide a general diagnosis that is relevant for all subjects in all clinical settings”^5^. Therefore, it is important to validate the new ESPEN criteria of malnutrition in elderly adults (especially in the elders living in nursing homes) because malnutrition occurs primarily in the elderly in developed countries^24^ and is highly prevalent in nursing homes^4^. Our study identified 23.1% participants had malnutrition defined by the new ESPEN criteria. A recent review summarized the relevant studies and demonstrated that the prevalence of malnutrition was approximately 20% in nursing homes. This finding was very similar to our results; however, none of these studies applied the new ESPEN criteria^4^.

The association between malnutrition and mortality in elderly adults has been well studied in various populations^4^. Recently, two studies also addressed the association between the new ESPEN definition of malnutrition and mortality in hospitalized patients^6,10^. Our study confirmed that the new ESPEN definition of malnutrition can predict mortality in nursing home residents for the first time. Therefore, our study adds new evidence to the application of the new ESPEN definition of malnutrition.

As a classic sarcopenia screening tool, SARC-F has been validated in various ethnic populations, such as Chinese, Korean, Japanese, and Spanish^25–29^. However, previous studies addressing SARC-F were conducted in community-dwelling elderly adults^25–29^. Our study supports the application of SARC-F in nursing home residents. SARC-F-defined sarcopenia can predict 1-year mortality in our study population. A previous study also found SARC-F-defined sarcopenia was associated with an increased risk of 4-year mortality in community-dwelling elderly adults^30^.

Previous studies have shown that malnutrition and sarcopenia are both common in elderly adults^4,21,31^. Our study revealed that both syndromes were prevalent in Chinese nursing home residents as well. Furthermore, as previously suggested, malnutrition and sarcopenia were also related geriatric syndromes in our study population. Because malnutrition and sarcopenia shared some risk factors (e.g., anorexia and aging) and clinical features (e.g., loss of body mass) and because they usually occur in individuals simultaneously^7,32^, Vandewoude and colleagues^32^ recently coined a term, malnutrition-sarcopenia syndrome (MSS), to describe the coexistence of malnutrition and sarcopenia. However, we did not apply the term of MSS in this study, because it has not been widely accepted yet.

Our study showed that coexisting malnutrition and sarcopenia can predict mortality in nursing home residents, but malnutrition without sarcopenia or sarcopenia without malnutrition cannot. This finding emphasized the importance of screening both malnutrition and sarcopenia simultaneously in the management of nursing home residents. The SARC-F is based on five self-reported questions. The new ESPEN definition of malnutrition is based on self-reported questions and BMI. Therefore, both tools are brief and suitable for nursing home residents. However, this finding needs to be further studies in other ethnic populations.

Some limitations of our study need to be addressed. First, the second ESPEN diagnostic criterion for malnutrition is based on the combination of unintentional weight loss, and low BMI or low fat-free mass index (FFMI)^5^. But we did not apply the FFMI in this study because the measurement of FFMI needs medical devices (e.g., bioelectrical impedance analyzer) that are generally unavailable in nursing homes. In fact, the new ESPEN consensus statement per se argued that “it was crucial not to mandate FFMI for the diagnosis of malnutrition”^5^. Second, the sample size of our study was relatively small, especially for those with coexisting malnutrition and sarcopenia. Third, our study was conducted in Chinese nursing home residents, therefore, it should be cautious when generalizing the results to other ethnic populations. Fourth, our study did not address the relationship between sarcopenia and malnutrition with disease-specific mortality. Last, like all cohort study, survival bias prior to entry in the cohort should be considered.

In conclusion, according to the new ESPEN criteria and SARC-F, malnutrition and sarcopenia are common in our study population and are independent predictors of mortality, respectively. When considering the two syndromes together, the coexistence of malnutrition and sarcopenia is a significant predictor of mortality, but malnutrition without sarcopenia or sarcopenia without malnutrition are not. Therefore, we suggest using brief and validated tools (like the new ESPEN definition and SARC-F) to assess malnutrition and sarcopenia simultaneously in nursing home residents. However, further prospective studies are certainly needed to validate this suggestion.

## Electronic supplementary material

Supplementary Tables

## References

[1] White JV (2012). Consensus statement: Academy of Nutrition and Dietetics and American Society for Parenteral and Enteral Nutrition: characteristics recommended for the identification and documentation of adult malnutrition (undernutrition). JPEN J Parenter Enteral Nutr.

[2] Guyonnet S, Rolland Y (2015). Screening for Malnutrition in Older People. Clin Geriatr Med.

[3] Saka B, Kaya O, Ozturk GB, Erten N, Karan MA (2010). Malnutrition in the elderly and its relationship with other geriatric syndromes. Clinical nutrition (Edinburgh, Scotland).

[4] Bell CL, Lee AS, Tamura BK (2015). Malnutrition in the nursing home. Curr Opin Clin Nutr Metab Care.

[5] Cederholm T (2015). Diagnostic criteria for malnutrition - An ESPEN Consensus Statement. Clinical nutrition (Edinburgh, Scotland).

[6] Rondel A, Langius JAE, de van der Schueren MAE, Kruizenga HM (2018). The new ESPEN diagnostic criteria for malnutrition predict overall survival in hospitalised patients. Clinical nutrition (Edinburgh, Scotland).

[7] Sanchez-Rodriguez D (2017). Prevalence of malnutrition and sarcopenia in a post-acute care geriatric unit: Applying the new ESPEN definition and EWGSOP criteria. Clinical nutrition (Edinburgh, Scotland).

[8] Ringaitiene Donata, Gineityte Dalia, Vicka Vaidas, Sabestinaite Akvile, Klimasauskas Andrius, Gaveliene Edita, Rucinskas Kestutis, Ivaska Justinas, Sipylaite Jurate (2018). Concordance of the new ESPEN criteria with low phase angle in defining early stages of malnutrition in cardiac surgery. Clinical Nutrition.

[9] Poulia KA (2017). The two most popular malnutrition screening tools in the light of the new ESPEN consensus definition of the diagnostic criteria for malnutrition. Clinical nutrition (Edinburgh, Scotland).

[10] Jiang J (2017). Predicting long-term mortality in hospitalized elderly patients using the new ESPEN definition. Sci Rep.

[11] Ingadottir Arora Ros, Beck Anne Marie, Baldwin Christine, Weekes C. Elizabeth, Geirsdottir Olof Gudny, Ramel Alfons, Gislason Thorarinn, Gunnarsdottir Ingibjorg (2018). Two components of the new ESPEN diagnostic criteria for malnutrition are independent predictors of lung function in hospitalized patients with chronic obstructive pulmonary disease (COPD). Clinical Nutrition.

[12] Dehesa-Lopez E, Martinez-Felix JI, Ruiz-Ramos A, Atilano-Carsi X (2017). Discordance between bioelectrical impedance vector analysis and the new ESPEN definition of malnutrition for the diagnosis of hospital malnutrition. Clin Nutr ESPEN.

[13] Sanz-Paris A (2016). Application of the new ESPEN definition of malnutrition in geriatric diabetic patients during hospitalization: A multicentric study. Clinical nutrition (Edinburgh, Scotland).

[14] Rojer AG (2016). The prevalence of malnutrition according to the new ESPEN definition in four diverse populations. Clinical nutrition (Edinburgh, Scotland).

[15] Marzetti E (2017). Sarcopenia: an overview. Aging-Clinical & Experimental Research.

[16] Cruz-Jentoft AJ (2010). Sarcopenia: European consensus on definition and diagnosis: Report of the European Working Group on Sarcopenia in Older People. Age Ageing.

[17] Chen LK (2014). Sarcopenia in Asia: consensus report of the asian working group for sarcopenia. J. Am. Med. Dir. Assoc..

[18] Fielding RA (2011). Sarcopenia: An Undiagnosed Condition in Older Adults. Current Consensus Definition: Prevalence, Etiology, and Consequences. International Working Group on Sarcopenia. J. Am. Med. Dir. Assoc..

[19] Studenski SA (2014). The FNIH Sarcopenia Project: Rationale, Study Description, Conference Recommendations, and Final Estimates. The Journals of Gerontology Series A: Biological Sciences and Medical Sciences.

[20] Malmstrom TK, Morley JE (2013). SARC-F: a simple questionnaire to rapidly diagnose sarcopenia. J Am Med Dir Assoc.

[21] Bauer JM, Kaiser MJ, Sieber CC (2008). Sarcopenia in nursing home residents. J Am Med Dir Assoc.

[22] Kaiser MJ (2009). Validation of the Mini Nutritional Assessment short-form (MNA-SF): a practical tool for identification of nutritional status. The journal of nutrition, health & aging.

[23] Lawton MP, Brody EM (1969). Assessment of older people: self-maintaining and instrumental activities of daily living. Gerontologist.

[24] Morley JE (2012). Undernutrition in older adults. Fam Pract.

[25] Parra-Rodriguez L (2016). Cross-Cultural Adaptation and Validation of the Spanish-Language Version of the SARC-F to Assess Sarcopenia in Mexican Community-Dwelling Older Adults. J Am Med Dir Assoc.

[26] Kim Sunyoung, Kim Miji, Won Chang Won (2018). Validation of the Korean Version of the SARC-F Questionnaire to Assess Sarcopenia: Korean Frailty and Aging Cohort Study. Journal of the American Medical Directors Association.

[27] Woo J, Leung J, Morley JE (2014). Validating the SARC-F: a suitable community screening tool for sarcopenia?. J Am Med Dir Assoc.

[28] Ida S (2017). Development of a Japanese version of the SARC-F for diabetic patients: an examination of reliability and validity. Aging Clin Exp Res.

[29] Ida, S. *et al*. Association Between Sarcopenia and Mild Cognitive Impairment Using the Japanese Version of the SARC-F in Elderly Patients With Diabetes. *Journal of the American Medical Directors Association* (2017). 10.1016/j.jamda.2017.06.012 28739493

[30] Wu TY (2016). Sarcopenia Screened With SARC-F Questionnaire Is Associated With Quality of Life and 4-Year Mortality. J Am Med Dir Assoc.

[31] Bruyere O (2016). Sarcopenia as a public health problem. Eur. Geriatr. Med..

[32] Vandewoude MF, Alish CJ, Sauer AC, Hegazi RA (2012). Malnutrition-sarcopenia syndrome: is this the future of nutrition screening and assessment for older adults?. Journal of Aging Research.

