# Supplementary material for: Applications of the new ESPEN definition of malnutrition and SARC-F in Chinese nursing home residents
Source: Sci Rep. 2018 Oct 8;8:14971. doi: 10.1038/s41598-018-33350-w (PMC6175895; doi:10.1038/s41598-018-33350-w)
Supplement: Supplementary file 1 — Supplementary Tables [file 41598_2018_33350_MOESM1_ESM.pdf]

Title page

Title:

Applications of the new ESPEN definition of malnutrition and SARC-F in Chinese nursing home residents

Authors:

Ming Yang, MD [1]; Zhaojing Huang, BSN [1]; Jing Chen, BS [1]; Jiaojiao Jiang, PhD [2]; Yun Zuo, BSN [3]; Qiukui Hao, MD [1]

Affiliations and addresses of all authors:

1. The Center of Gerontology and Geriatrics, West China Hospital, Sichuan University, No. 37 Guoxue Lane, Chengdu, Sichuan, China.
2. The Health Management Center, Shangjin Nanfu Hospital, No. 253 Shangjin Street, Chengdu, Sichuan, China.
3. The Center of Rehabilitation, West China Hospital, Sichuan University, No. 37 Guoxue Lane, Chengdu, Sichuan, China.

Information of the corresponding author:

Ming Yang, The Center of Gerontology and Geriatrics, West China Hospital, Sichuan University, No. 37 Guoxue Lane, Chengdu, China. Phone: +86 28 8542 2326. Fax: +86 28 8542 2321. Email: yangmier@gmail.com

Supplementary Table 1. The SARC-F scale. Adapted from Malmstrom et al. [1]

Components	Questions	SARC-F Score
Strength	How much difficulty do you have in lifting and carrying 10 pounds?	None=0<br>Some =1<br>A lot or unable = 2
Assistance in walking	How much difficulty do you have walking across a room?	None=0<br>Some =1<br>A lot, use aids, or unable = 2
Rise from a chair	How much difficulty do you have transferring from a chair or bed?	None=0<br>Some =1<br>A lot or unable without help = 2
Climb stairs	How much difficulty do you have climbing a flight of 10 stairs?	None=0<br>Some =1<br>A lot or unable = 2
Falls	How many times have you fallen in the past year?	None=0<br>1-3 falls =1<br>4 or more falls = 2

References

1. Malmstrom TK, Morley JE. SARC-F: a simple questionnaire to rapidly diagnose sarcopenia. J Am Med Dir Assoc 2013;14(8):531-2.

Supplementary Table 2. Baseline characteristics of the study population according to malnutrition and sarcopenia separately

Characteristic	SARC-F			ESPEN definition		
	Without sarcopenia (n=198)	Sarcopenia (n=131)	P	Without malnutrition (n=253)	malnutrition (n=76)	P
Age (years)*	84.0 (2.0)	85.0 (3.0)	0.016	84.0 (3.0)	85.0 (3.0)	0.013
Women (%)	126 (63.6)	98 (74.8)	0.033	169 (66.8)	55 (72.4)	0.361
Current smokers (%)	7 (3.5)	3 (2.3)	0.745	7 (2.8)	3 (3.9)	0.599
Current alcohol drinkers (%)	1.4 (7.1)	9 (6.9)	0.944	20 (7.9)	3 (3.9)	0.235
Comorbidities (%)						
Hypertension	56 (28.3)	43 (32.8)	0.379	85 (33.6)	14 (18.4)	0.011
Ischemic heart disease	14 (7.1)	19 (14.5)	0.028	27 (10.7)	6 (7.9)	0.480
CHF	63 (31.8)	49 (37.4)	0.295	86 (34.0)	26 (34.2)	0.972
COPD	21 (10.6)	22 (16.8)	0.103	36 (14.2)	7 (9.2)	0.255
Diabetes	22 (11.1)	11 (8.4)	0.422	28 (11.1)	5 (6.6)	0.253
Stroke	39 (19.7)	17 (13.0)	0.112	46 (18.2)	10 (13.2)	0.307
Cancer	16 (8.1)	10 (7.6)	0.883	23 (9.1)	3 (3.9)	0.145
Osteoarthritis	108 (54.5)	83 (63.4)	0.113	147 (58.1)	44 (57.9)	0.974
Parkinson's disease	10 (5.1)	21 (16.0)	0.001	23 (9.1)	8 (10.5)	0.707
Cognitive impairment	48 (24.2)	23 (17.6)	0.149	49 (19.4)	22 (28.9)	0.075
Depression	36 (18.2)	32 (24.4)	0.171	54 (21.3)	14 (18.4)	0.581
Nutritional supplements	28 (14.1)	23 (17.6)	0.402	39 (15.4)	12 (15.8)	0.937
SARC-F score *	2.0 (2.0)	4.0 (1.0)	<0.001	3.0 (2.0)	3.5 (3.0)	0.039
MNA-SF score *	13.0 (2.5)	11.0 (3.0)	0.025	13.0 (3.0)	10.0 (2.5)	<0.001
BMI (women, kg/m^2) *	24.2 (3.9)	23.9 (4.3)	0.733	25.1 (3.5)	22.0 (2.5)	<0.001
BMI (men, kg/m^2) *	24.1 (4.6)	23.0 (4.9)	0.092	24.7 (4.0)	20.0 (1.7)	<0.001

CC (women, cm) *	32.0 (4.0)	32.0 (3.0)	0.047	32.0 (3.0)	30.0 (3.0)	<0.001
CC (men, cm) *	33.0 (4.0)	32.0 (4.0)	0.005	33.0 (3.0)	30.0 (4.0)	<0.001
WC (women, cm) *	85.0 (12.0)	85.0 (13.0)	0.840	87.0 (10.0)	76.0 (10.0)	<0.001
WC (men, cm) *	86.0 (12.0)	82.0 (17.5)	0.210	87.0 (12.5)	75.0 (10.0)	<0.001
ADL disability (%)	74 (37.4)	64 (48.9)	0.884	99 (39.1)	39 (51.3)	0.059

* Data are presented as median (IQR).

ADL: activities of daily living; BMI: body mass index; CC: calf circumference; CHF: chronic heart failure; COPD: chronic obstructive pulmonary disease; IQR: interquartile range; MNA-SF: Mini Nutritional Assessment Short-Form; MN+/SA+: malnutrition combined with sarcopenia; MN+/SA-: malnutrition without sarcopenia; MN-/SA+: normal nutrition with sarcopenia; MN-/SA-: normal nutrition without sarcopenia; WC: waist circumference.
